# Natural killer cell therapy potentially enhances the antitumor effects of bevacizumab plus irinotecan in a glioblastoma mouse model

**DOI:** 10.3389/fimmu.2022.1009484

**Published:** 2023-01-10

**Authors:** Thi-Anh-Thuy Tran, Young-Hee Kim, Thi-Hoang-Oanh Duong, JayaLakshmi Thangaraj, Tan-Huy Chu, Shin Jung, In-Young Kim, Kyung-Sub Moon, Young-Jin Kim, Tae-Kyu Lee, Chul Won Lee, Hyosuk Yun, Je-Jung Lee, Hyun-Ju Lee, Kyung-Hwa Lee, Tae-Young Jung

**Affiliations:** ^1^ Brain Tumor Research Laboratory, Chonnam National University Hwasun Hospital, Hwasun, Republic of Korea; ^2^ Biomedical Sciences Graduate Program (BMSGP), Chonnam National University Medical School, Hwasun, Republic of Korea; ^3^ Research Center for Cancer Immunotherapy, Chonnam National University Hwasun Hospital, Hwasun, Republic of Korea; ^4^ Department of Neurosurgery, Chonnam National University Medical School, and Hwasun Hospital, Hwasun, Republic of Korea; ^5^ Department of Chemistry, Chonnam National University, Gwangju, Republic of Korea; ^6^ Department of Internal Medicine, Chonnam National University Medical School, and Hwasun Hospital, Hwasun, Republic of Korea; ^7^ Department of Pathology, Chonnam National University Medical School, and Hwasun Hospital, Hwasun, Republic of Korea

**Keywords:** natural killer cells, bevacizumab, irinotecan, U87 cell line, glioblastoma

## Abstract

Various combination treatments have been considered to attain the effective therapy threshold by combining independent antitumor mechanisms against the heterogeneous characteristics of tumor cells in malignant brain tumors. In this study, the natural killer (NK) cells associated with bevacizumab (Bev) plus irinotecan (Iri) against glioblastoma multiforme (GBM) were investigated. For the experimental design, NK cells were expanded and activated by K562 cells expressing the OX40 ligand and membrane-bound IL-18 and IL-21. The effects of Bev and Iri on the proliferation and NK ligand expression of GBM cells were evaluated through MTT assay and flow cytometry. The cytotoxic effects of NK cells against Bev plus Iri-treated GBM cells were also predicted *via* the LDH assay *in vitro*. The therapeutic effect of different injected NK cell routes and numbers combined with the different doses of Bev and Iri was confirmed according to tumor size and survival in the subcutaneous (s.c) and intracranial (i.c) U87 xenograft NOD/SCID IL-12Rγ^null^ mouse model. The presence of injected-NK cells in tumors was detected using flow cytometry and immunohistochemistry *ex vivo*. As a result, Iri was found to affect the proliferation and NK ligand expression of GBM cells, while Bev did not cause differences in these cellular processes. However, the administration of Bev modulated Iri efficacy in the i.c U87 mouse model. NK cells significantly enhanced the cytotoxic effects against Bev plus Iri-treated GBM cells *in vitro.* Although the intravenous (IV) injection of NK cells in combination with Bev plus Iri significantly reduced the tumor volume in the s.c U87 mouse model, only the direct intratumorally (IT) injection of NK cells in combination with Bev plus Iri elicited delayed tumor growth in the i.c U87 mouse model. Tumor-infiltrating NK cells were detected after IV injection of NK cells in both s.c and i.c U87 mouse models. In conclusion, the potential therapeutic effect of NK cells combined with Bev plus Iri against GBM cells was limited in this study. Accordingly, further research is required to improve the accessibility and strength of NK cell function in this combination treatment.

## 1 Introduction

Glioblastoma multiforme (GBM) is the most frequent and aggressive type of cancer in the central nervous system (CNS). It is associated with poor prognostic factors and low survival. It accounts for 14.3% of all primary brain and other CNS tumors and 49.1% of primary malignant brain tumors. Only 6.8% of patients survive after 5 years of diagnosis (1, 2). Although advances in GBM treatments, e.g., maximal surgical resection with radiation and chemotherapy, have been applied, the mean 5-year overall survival (OS) of patients with GBM remains low (approximately 9.8%) (3, 4). Therefore, new strategies must be developed to improve the therapeutic effects of this disease.

GBM is a highly vascularized tumor that secretes a large amount of vascular endothelial growth factor (VEGF) (5). VEGF is a key mediator of tumor neovascularization (endothelial proliferation and vascular permeability) that suppresses most immune responses in tumors (6–9). As a standard treatment for GBM, bevacizumab (Bev) is a humanized monoclonal antibody approved by the FDA. It targets the VEGF receptor and consequently reduces blood flow and tumor volume (10). Furthermore, irinotecan (Iri)—a chemotherapeutic drug—is found to be effective with tumor cells by blocking a topoisomerase 1 inhibitor involved in the induction of DNA damage and subsequent apoptosis. It can cross the blood-brain barrier and exhibits unique antitumor activity against GBM as observed in preclinical and clinical investigations (11–13).

Recent clinical trials using Bev in combination with Iri and/or temozolomide in recurrent patients with GBM have demonstrated its moderate effectiveness and tolerance (14, 15). Bev combined with Iri is effective for patients with recurrent malignant gliomas and some children with recurrent low-grade gliomas. This combination is safe and has excellent activity even in the relapsed and heavily pretreated population of patients with malignant glioma, most of whom were not candidates for clinical trials (16, 17). The efficacy of Bev plus Iri can be attributed to the antitumor stem cell effect of Bev, the anti-differentiated glioma tumor cell effect of Iri, and the normalization of tumor vasculature; consequently, interstitial pressure decreases, hypoxia is minimized, and the delivery of Iri to tumors is enhanced (18, 19).

Although natural killer (NK) cells in patients with cancer are often functionally compromised owing to the immunosuppressive activity of tumors, the initial success of adoptive NK cell transfer in the treatment of hematological cancers has motivated clinical efforts devoted to using this strategy against solid cancers. NK cells not only elicit cytotoxic effects against a wide range of tumor cells of solid cancer types but also exhibit antitumor activities in preclinical xenograft mouse models of GBM. The safety of NK cell-based therapy has been evaluated in autologous and allogeneic haploidentical settings. However, the clinical efficacy of this strategy is limited (20–22). Therefore, the combination of NK cells with other treatment methods in current preclinical efforts enhances the efficacy of NK cell-based therapy. Furthermore, NK cells expanded and activated by K562-OX40L-mb-IL-18/IL-21 feeder cells have shown cytotoxic activity against multiple myeloma in *in vitro* and xenograft mouse models (23–25). Therefore, NK cells using K562-OX40L-mb-IL-18/IL-21 cells as feeder cells were also used to confirm therapeutic effects against GBM in this study.

Tumor cell heterogeneity is a crucial characteristic that contributes to therapeutic resistance and the recurrence of malignant brain tumors (26). Individual treatments may have a limited effect; however, combined treatments may achieve the threshold for effective therapy. Although the therapeutic effects of Bev plus Iri have been demonstrated, the role of the combination of Bev and Iri in NK therapy has not been investigated. NK cells, Bev, and Iri are known to have different working mechanisms toward target tumors (11, 27, 28). Nevertheless, more complicated possible interactions between NK cells, Bev, and Iri should be confirmed for improving their anti-GBM effects. In this study, NK cells were used in combination with Bev and Iri to further understand the preclinical success and aid in designing rational and effective combinatorial therapies for GBM in clinical settings.

## 2 Materials and methods

### 2.1 Patient samples and animals

Blood and tissue samples were collected from healthy donors (HDs) and GBM patients with the approval of the institutional ethical committee at Chonnam National University Hwasun Hospital. All surgical tissues and blood from GBM patients were collected at the Neurosurgery Department at Chonnam National University Hwasun Hospital.

Six-to-eight-week-old female NOD/SCID IL-12Rγ^null^ (NSG) mice (Jackson Laboratory, Bar Harbor, MA, USA) were raised under specific pathogen-free conditions. The mice were anesthetized by the intraperitoneal injection (IP) of a 2:1 mixture of Zoletil^®^ (Virbac Laboratories, Carros, France)/Rompun^®^ (Bayer Korea, Anshan, Korea) at a dose of 1.5 mL/kg. All animal care procedures, experiments, and euthanasia were performed after obtaining approval from the Chonnam National University Animal Research Committee.

### 2.2 Cell lines

Human GBM cell lines, namely, U87, U118, and U343, were obtained from the Korean Cell Line Bank (Seoul, Korea) and the Brain Tumor Research Center, University of California, San Francisco. All cells were maintained in Dulbecco’s Modified Eagle’s Medium (DMEM) supplemented with 10% heat-inactivated fetal bovine serum (FBS; Gibco, US) and 1% penicillin–streptomycin in a humidified 5% CO_2_ incubator at 37°C.

Human K562 (human immortalized myelogenous leukemia cell line) was purchased from the American Type Culture Collection (ATCC, Manassas, VA, USA). Modified K562-OX40L-mb-IL-18/IL-21 cells were used to expand and activate NK cells (25). Cells were cultured in Roswell Park Memorial Institute (RPMI)-1640 medium supplemented with 10% heat-inactivated FBS (Gibco, US) and 1% penicillin–streptomycin in a humidified 5% CO_2_ incubator at 37°C.

### 2.3 Chemical agents and flow cytometry

Bev (Avastin^®^) and Iri-HCl (Campto^®^) were purchased from Roche (Basel, Switzerland) and Inno.N (Seoul, Republic of Korea) laboratories, respectively. Dose treatments were set at 10 mg/kg Bev plus 125 mg/m^2^ Iri-HCl (a high dose of Bev and Iri, Bev plus Iri^high^) and 5 mg/kg Bev plus 60 mg/m^2^ Iri (a low dose of Bev and Iri, Bev plus Iri^low^) according to the previously reported clinical trial dose (29–32). The dose was converted from a human dose into a mouse dose for treatment (33). The abovementioned chemical agents were administered through IP.

The expression levels of NK ligands on GBM cells, NK markers on NK cells, and NK purity were confirmed through flow cytometry. The cells were stained with antibodies listed in **Table 1**. The mean fluorescence intensity (MFI) ratio was calculated by dividing the MFI of the stained cell population by that of the unstained cell population. Fluorescence minus one (FMO) stain was used in each sample in each experiment to determine positive and negative populations. Further, gates were applied from FMOs to samples. The FMO of each experiment has been described in the corresponding figure legends.

**Table 1:** List of antibodies used in flow cytometry.

Name	Catalog#	Clone	Company
LIVE/DEAD™ Fixable Dead Cell Stain Kits	L34966		Invitrogen
PE-Mouse anti-human CD226 (DNAM1)	559789	DX11 (RUO)	BD PhanningenTM
APC-Mouse anti-human CD45	555485	HI30 (RUO)	BD PhanningenTM
FITC Mouse Anti-human CD3	555332	UCHT1	BD PhanningenTM
APC- Mouse Anti-human CD56	55518	B159	BD PhanningenTM
PE-Mouse anti-human CD178 (FASL)	564261	NOK-1 (RUO)	BD PhanningenTM
PE-Mouse anti-human CD253 (TRAIL)	550516	RIK-2 (RUO)	BD PhanningenTM
PE-anti-human PD1	FAB7115P	Polyclonal Goat IgG	R&D systems
PE-Mouse anti-human CD337 (NKp30)	558407	p30-15 (RUO)	BD PhanningenTM
PE- anti-human NKG2A/CD159a	FAB1059P	Monoclonal Mouse lgG2A Clone # 131411	R&D systems
PE Mouse anti-Human CD314 (NKG2D)	557940	Monoclonal (1D11)	BD PhanningenTM
APC Mouse Anti-Human CD274 (PDL1)	563741	MIH1 (RUO)	BD PhanningenTM
APC anti-human ULBP-1	FAB1380A	Monoclonal Mouse lgG2A Clone # 170818	R&D systems
APC anti-human MICA/MICB	320908	6D4	BioLegend
APC anti-human CD112 (Nectin-2)	337412	TX31	BioLegend
APC anti-human CD155 (PVR)	337618	SKII.4	BioLegend

### 2.4 3-(4,5-Dimethylthiazol-2-yl)-2,5-diphenyltetrazolium bromide (MTT) cell viability assay

The effects of Bev and Iri on the proliferation and survival of GBM cells (U87, U118, and U343 cells) were predicted *via* the MTT assay. Briefly, GBM cells (3×10^3^ cells/well) were seeded in 96-well plates (SPL, Gyeonggi-do, Korea) and cultured with DMEM supplemented with 10% FBS and 1% P/S at 37°C in a 5% CO_2_ atmosphere. They were stained after 24 h and 48 h incubation with MTT (Sigma). For staining, the plates were washed with PBS, and MTT (0.5 mg/mL) was added to each well. After 4 h of incubation, the MTT solution was removed from each well. MTT formazan was then solubilized using isopropanol (Merck, Darmstadt, Germany), and optical density was read at 570 nm.

### 2.5 NK cell culture

NK cells were cultured and expanded using the established K562-OX40L-mb-IL-18/IL-21 feeder cells in previously described methods (25). Briefly, peripheral blood mononuclear cells isolated from HDs and GBM patients were co-cultured with gamma-irradiated (100 Gy) K562-OX40L-mb-IL-18/IL-21 feeder cells in RPMI-1640 medium containing 10% FBS, 1% penicillin/streptomycin, and 4 mM L-glutamine. NK cells were cultured in the presence of recombinant human interleukin IL-2 and IL-15 (PeproTech, Rocky Hill, NJ, USA). The recombinant IL-2 (10 U/mL) was added to the cell culture medium until day 7. From day 7, recombinant IL-2 (100 U/mL) and recombinant IL-15 (5ng/mL) were added to the cell culture medium. The cell culture medium containing cytokines was refreshed every 2–3 days. On day 14, NK cells with >90% purity were used for *in vitro* and *in vivo* experiments. For *in vivo* experiments, NK cells were expanded from isolated peripheral blood mononuclear cells of HDs (NK-HD cells) used for all experiments. The mice were treated with intravenously injected NK-HD cells at a low dose (1×10^7^ NK cells/ injection, NK-IV^low^) or a high dose (2×10^7^ NK cells/ injection, NK-IV^high^), and intratumorally injected NK-HD cells (2×10^6^ NK cells/ injection, NK-IT), respectively.

### 2.6 Lactate dehydrogenase release cytotoxicity assay

CytoTox 96 nonradioactive cytotoxicity assay (CytoTox 96, Promega, Madison, WI, USA) was performed to analyze the cytotoxic effects of NK-HD and NK-GBM cells against target GBM cells, namely, U87, U118, U343, and primary GBM (pGBM) cells, according to the manufacturer’s instructions. Briefly, GBM cells (4×10^4^ cells/ well) with or without treatment with Bev and Iri were used as the target. The NK cells were co-cultured with the target cells at 1:1 and 1:3 (target: effector) ratio in Costar 96-well plates (SPL, Gyeonggi-do, Korea) under a 5% CO_2_ atmosphere at 37°C for 5 h. Then, supernatants were collected to determine the LDH concentration. The mean percentage of specific lysis was calculated as follows:


(1) $$\text{\% Cytotoxicity}=\frac{\left(\text{Experimental }-\text{ Effector Spontaneous }-\text{ Target Spontaneous}\right)}{\left(\text{Target Maximum }-\text{ Target Spontaneous}\right)}\times 100$$

### 2.7 Enzyme-linked immunosorbent assay

The GBM cells U87, U118, U343, and pGBM (4 × 10^4^ cells/wells) with or without treatment with Bev and Iri were used as target cells. The NK-HD and NK-GBM cells were co-cultured with target cells at a 1:3 (target:effector) ratio in 96-well plates (SPL, Gyeonggi-do, Korea) under a 5% CO_2_ atmosphere at 37°C for 5 h. Subsequently, the supernatants were collected to determine the IFN-γ concentration by using the OptEIA ELISA kit (BD Bioscience) as per the manufacturer’s instructions. NK alone and NK treated with dimethyl sulfoxide (DMSO) were used as the negative control and NK treated with PMA/Ionomycin (Biolegend, 423302) was used as the positive control for released IFN-γ.

### 2.8 Subcutaneous and intracranial U87 xenograft mouse model

For the s.c mouse model, 5×10^6^ U87 cells in 100 µl of PBS were mixed with 100 µl of Matrigel™ Matrix (Corning #354248) and injected into the right s.c of NSG mice. The mice were randomly allocated to the treatment arms. NK-HD cells were injected intravenously (IV). For the treatment, the mice were divided into four treatment groups: (1) control, (2) NK-IV^low^, (3) Bev plus Iri^high^, and (4) NK-IV^low^ combined with Bev plus Iri^high^. The treatment was initiated when the tumor size reached approximately 100 mm^3^. Overall survival was quantified, and the tumor volume was calculated using the standard formula for the volume of an ellipsoid: V=4/3π [(length × width × height)/8]. Once the total tumor volume reached 1000 mm^3^ per animal, the mice were euthanized.

For the i.c mouse model, 5×10^5^ U87 cells in 5 μl of PBS were stereotactically injected into the right striatum of the mice at a rate of 1 μl/min. Injection sites were estimated using the following coordinates: 1 mm posterior, 2 mm lateral from the bregma, and 4 mm deep from the cortical surface. The mice were randomly allocated to the treatment arms. NK-HD cells were injected IV or intratumorally (IT). For the treatment, the mice were divided into eight and four different treatment groups. For the eight groups, the mice were treated according to (1) control, (2) NK-IV^low^, (3) NK-IT, (4) Bev plus Iri^low^, (5) NK-IV^high^ combined with Bev plus Iri^low^, (6) Bev plus Iri^high^, (7) NK-IV^low^ combined with Bev plus Iri^high^, and (8) NK-IT combined with Bev plus Iri^high^. For the four groups, the mice were treated according to (1) control, (2) Iri^high^, (3) Bev plus Iri^high^, and (4) NK-IV^high^ combined with Bev plus Iri^high^. The treatments were initiated when the tumor size reached approximately 5 mm^3^. The overall survival was quantified. Tumor size was confirmed through magnetic resonance imaging (MRI) and analyzed using the RadiAnt DICOM Viewer 2021.2 software. Tumor volume was calculated by the summation of all tumor areas in each slide and multiplication by the slide thickness.

### 2.9 Tumor dissociation

Single tumor cells were collected from GBM patients and U87-bearing NSG mice were collected for further experiment. Single tumor cells from GBM patients were used for confirming NK ligands expression and target cells of cytotoxicity assay, while those from the U87-bearing NSG mice were used for confirming the presence of NK cells in the tumor and NK ligand expression on single tumor cells before and after treatment with NK cells, Bev, and Iri. The single tumor cells were collected according to the following protocol. First, the tumor was collected, washed with RPMI media supplemented with 10% FBS and 1% P/S, and minced into 3 mm to 4 mm pieces by using a sterile scalpel. Then, the tumor was dissociated by using a tumor dissociation kit (Miltenyi Biotech, Germany) for obtaining the single tumor cells from the s.c tumor and a brain tumor dissociation kit (Miltenyi Biotech, Germany) for obtaining the single tumor cells from the i.c tumor. Afterward, the cells were filtered using 70 or 40 μm cell strainers (Falcon), and single tumor cells were collected. Erythrocytes were removed using red blood cell lysis buffer (Miltenyi Biotech, Germany).

### 2.10 Immunohistochemistry

Tumors from the s.c and i.c mouse models were collected and fixed in 5% paraformaldehyde to confirm the presence of NK cells at tumor sites. They were then embedded in paraffin, sectioned into 4 μm coronal sections by using a microtome, and prepared for immunohistochemistry (IHC) staining. The antibodies used for the IHC experiment were anti-human CD56 (1:50 dilution; Code No. M7304; clone 123C3; DAKO; Denmark) and anti-human CD45RO (LCA; 1:100 dilution; Code No. M0701; clone 2B11+PD7/26; DAKO; Denmark). Tumor slides were scanned using the Aperio Scan Scope System (Aperio, Technology, Vista, CA, USA).

### 2.11 Statistical analysis

Data were statistically analyzed using SPSS 23.0 for Windows (SPSS Inc., Chicago, IL, USA). One-way or two-way ANOVA was performed for analyses across multiple groups. A log-rank test was performed on survival data with Bonferroni correction applied for comparations, and an independent sample t-test was used to compare significant differences between the two groups. Data with p<0.05 were considered statistically significant.

## 3 Results

### 3.1 Effects of Bev and Iri on the proliferation of GBM cells

**Figure 1:**
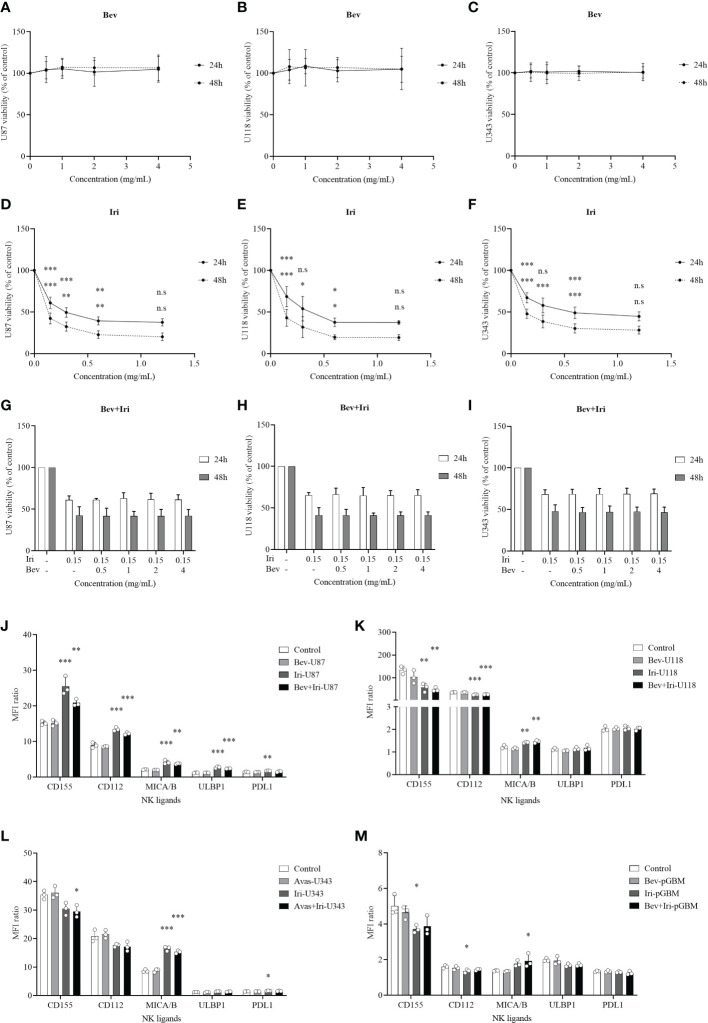
Effects of Bev and Iri on the proliferation of GBM cells *in vitro*. GBM cells (U87, U118, and U343 cells) were treated with Bev and Iri separately or in combination of according to the increased concentration of these two drugs (0.5, 1, 2, and 4 mg/mL for Bev and 0.15, 0.3, 0.6, and 1.2 mg/mL for Iri). After 24 and 48 h, cell viability was determined by MTT assay and IC_50_ was calculated to determine the concentration of drugs that exhibited 50% cell viability. The viability of untreated cell cultures was set at 100% **(A–I)**. Moreover, the expression of NK ligands on GBM cells such as U87, U118, U343, and pGBM and the effects of Bev (0.5 mg/mL) and Iri (0.15 mg/mL) on their expression was clarified using flow cytometry. **(J–M)** Data were presented as MFI ratio calculated by dividing the MFI of the positive cells (stained cell population) by that of the negative cells (live cell population). FMO: live cells. U87, U118, and U343: human GBM cell lines; pGBM: primary GBM cells; Bev: bevacizumab; Iri: irinotecan. All data are shown as mean ± SD. p< 0.05 (*), p< 0.001 (**), p< 0.0001 (***). n.s, no significant difference.

The viability percentages and IC_50_ of GBM cells (U87, U118, and U343 cells) after the treatment with Bev and Iri separately or in combination with a gradual increase in dose concentrations (0.5, 1, 2, and 4 mg/mL for Bev and 0.15, 0.3, 0.6, and 1.2 mg/mL for Iri) were determined at 24 and 48 h *via* the MTT assay to investigate the effects of Bev and Iri on the proliferation of GBM cells (**Figures 1A–I**).

Bev did not affect the viability of GBM cells, whereas Iri significantly decreased the viability of all GBM cells according to the increased dose statuses (0.15, 0.3, 0.6, and 1.2 mg/mL) at 24 and 48 h. In particular, 0.15 mg/mL Iri reduced 37% (p=0.000, 24 h) and 55% (p=0.000, 48 h) of U87 cell viability, 31% (p=0.000, 24 h) and 57% (p=0.000, 48 h) of U118 cell viability, and 33% (p=0.000, 24 h) and 52% (p=0.000, 48 h) of U343 cell viability compared with those in the control group. This trend was similar to 0.3 mg/mL and 0.6 mg/mL Iri. However, after 24 and 48 h, GBM cell viability did not vary between 0.6 mg/mL and 1.2 mg/mL Iri. Thus, the examined GBM cells might exhibit resistance at an Iri concentration of 1.2 mg/mL.

Iri exhibited 50% cell viability (IC_50_) at 0.33 mg/mL and 0.08 mg/mL for U87 cells, 0.41 mg/mL and 0.09 mg/mL for U118 cells, and 0.63 mg/mL and 0.11 mg/mL for U343 cells at 24 h and 48 h of incubation, respectively. Because Bev did not affect GBM cell viability, the minimal screening Iri dose of 0.15 mg/mL that significantly affected GBM cell proliferation but had not reached resistance on GBM cells was combined with different doses of Bev (0.5, 1, 2, and 4 mg/mL) to confirm GBM cell viability. Results of the analysis revealed that the GBM cell viability did not significantly differ among these combinations. Therefore, the screened dose of 0.5 mg/mL Bev and 0.15 mg/mL Iri were used for further *in vitro* analysis.

### 3.2 Effects of Bev and Iri on the NK ligand expression of GBM cells

The expression levels of NK ligands (CD155, CD112, MICA/B, ULBP1, and PDL1) on GBM cells (U87, U118, and U343) and primary GBM cells (pGBM) before and after the treatments with Bev (0.5 mg/mL) and Iri (0.15 mg/mL) at 24 h were elucidated *via* flow cytometry (**Figures 1J–M**; **S1**). Although Iri affected the NK ligands on GBM cells, Bev did not influence their expression. In particular, the MICA/B expression was enhanced under Iri or Bev plus Iri treatment in U87 cells (p=0.000 and p=0.001), U118 cells (p=0.007 and p=0.003), and U343 cells (p=0.000 and p=0.000) compared control group. However, only Bev plus Iri increased MICA/B expression in pGBM cells (p = 0.05) compared to the control group. CD155 and CD112 expression levels increased in U87 cells with Iri (p = 0.000 and p = 0.000) and Bev plus Iri group (p = 0.007 and p = 0.000), while it decreased in U118 cells with Iri (p = 0.006 and p = 0.000) and Bev plus Iri group (p = 0.003 and p = 0.000). Furthermore, while Bev plus Iri reduced CD155 expression on U343 cells (p = 0.03), Iri caused a reduction in CD155 and CD122 in pGBM cells (p = 0.033 and p = 0.034, respectively). Moreover, only U87 cells exhibited an increase in ULBP1 expression under Iri and Bev plus Iri treatment (p = 0.000 and p = 0.000, respectively). Therefore, the synergistic effects of Iri and NK cells could be related to the MICA/B expression; however, further studies should be performed to verify this finding.

### 3.3 NK cell characterization

The schematic of NK cell expansion from peripheral blood mononuclear cells (PBMCs) of HDs (NK-HD) and GBM patients (NK-GBM) co-cultured with 100 Gy gamma-irradiated K562-OX40L-mb-IL-18/IL-21 feeder cells in the presence of IL-2 (from day 0 to day 6) and IL-2/IL-15 (from day 7 to day 28) is shown in **Figure S2A**. The NK-HD and NK-GBM cells were characterized by NK purity, expansion fold, and NK marker expression. In particular, the NK-HD and NK-GBM cells were confirmed in terms of NK purity, expansion fold, and NK marker expression every 7 days (on days 0, 7, 14, 21, and 28) (**Figures S2B–D**). There was no difference between the observed NK-HD and NK-GBM cells.

NK-HD and NK-GBM cell purity did not vary on days 14, 21, and 28. Although the expansion fold of NK-HD cells was higher than that of NK-GBM cells (around 4000-fold expansion for NK-HD and 3000-fold expansion for NK- GBM), it did not significantly differ between NK-HD and NK-GBM cells for 28 days. Moreover, the expression of NK markers from NK-HD and NK-GBM cells was estimated every 7 days (on days 0, 7, 14, 21, and 28). The activated and inhibited NK marker expression (NKG2D, NKp30, FASL, TRAIL, DNAM1, PD1, and NKG2A) did not differ among days 0, 7, 14, 21, and 28; similarly, the NK marker expression did not vary between NK-HD and NK-GBM cells.

### 3.4 Cytotoxic function of NK cells against GBM cells *in vitro*

**Figure 2:**
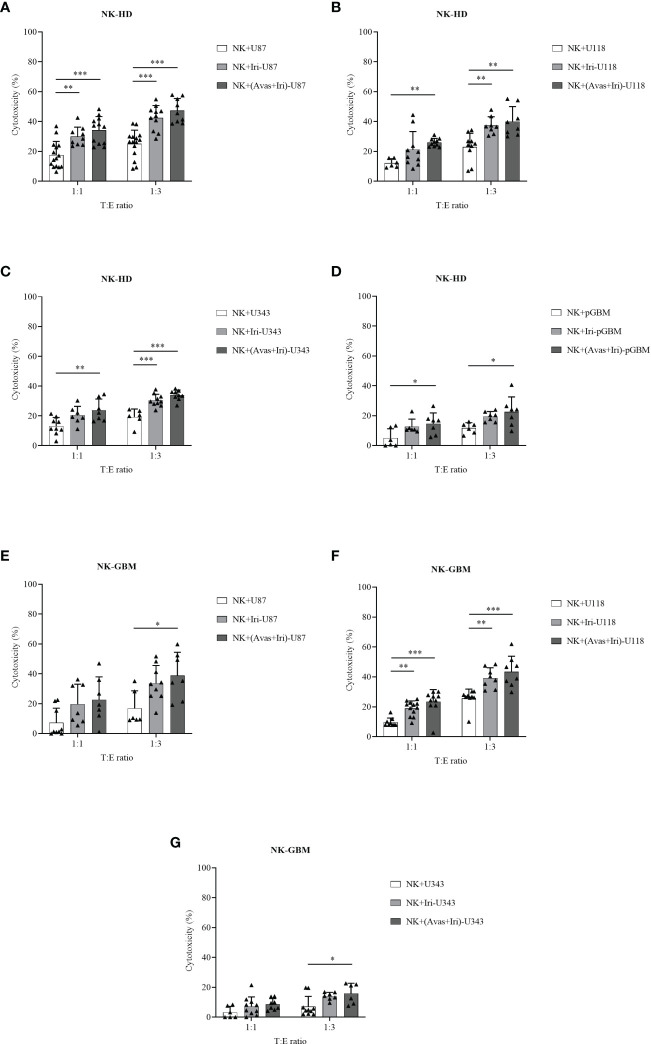
Cytotoxic effects of expanded NK cells from HDs (NK-HD) and GBM patients (NK-GBM) against GBM cells, Iri-treated GBM, and Bev plus Iri-treated GBM cells. The cytotoxicity effects of NK-HD **(A–D)** and NK-GBM cells **(E–G)** against GBM cells such as U87, U118, U343, pGBM at 1:1 and 1:3 (target:effector) ratio were confirmed through the LDH assay. U87, U118, and U343: human GBM cell lines; pGBM: primary GBM cells; Bev: bevacizumab (0.5 mg/mL); Iri: irinotecan (0.15 mg/mL). All data are shown as mean ± SD. p< 0.05 (*), p< 0.001 (**), p< 0.0001 (***).

The cytotoxic effects of NK-HD and NK-GBM cells against GBM cell lines (U87, U118, and U343) and pGBM cells were estimated in **Figure 2**. Generally, NK-HD and NK-GBM cells exhibited cytotoxic effects against all GBM cells (U87, U118, U343, and pGBM) at 1:1 and 1:3 (target:effector) ratio. Although both NK-HDs and NK-GBM enhanced the cytotoxic effects against GBM cells treated with Bev plus Iri compared to the untreated group, no difference was observed in the cytotoxicity of NK-HDs and NK-GBM against Iri-treated GBM cells and Bev plus Iri-treated GBM cells. Thus, the main effects that enhanced NK cytotoxicity against GBM cells *in vitro* could be related to Iri rather than Bev. This trend was observed clearly at a 1:3 (target:effector) ratio. Particularly, the cytotoxicity of NK-HD cells against GBM cells treated with Bev plus Iri was enhanced by 22% (p = 0.000) in U87 cells, 17% (p = 0.001) in U118 cells, 14% (p = 0.000) in U343 cells, and 11% (p = 0.022) in pGBM cells compared to those in the untreated group. Similarly, the cytotoxicity of NK-GBM cells against GBM cells treated with Bev plus Iri was enhanced by 22% (p = 0.02) in U87 cells, 17% (p = 0.000) in U118 cells, and 9% (p = 0.024) in U343 cells compared to those in the untreated group. However, the cytotoxicity of NK cells against GBM cells treated with Iri alone and Bev plus Iri showed no significant difference between NK-HD and NK-GBM cells, except in U343 cells.

The IFN-γ levels before and after co-culturing with untreated GBM cells, Iri-treated GBM cells, and Bev plus Iri-treated GBM cells can be confirmed from **Figure S3**. In particular, the IFN-γ levels of NK-HDs and NK-GBM cells were enhanced after co-culturing with GBM target cells. Although the IFN-γ levels of NK-HDs and NK-GBM against Iri- and Bev plus Iri-treated GBM target cells showed a slight enhancement compared to untreated GBM target cells, no difference was observed in the IFN-γ levels between Iri- and Bev plus Iri-treated GBM group. Further, the expression of NK cell markers (NKG2D, NKp30, FASL, TRAIL, DNAM1, NKG2A, and PD1) from NK-GBM cells before and after co-culturing with the untreated (U87, U118, and U343 cells), Iri-treated, and Bev plus Iri-treated GBM cells are illustrated in **Figure S4**. No significant differences were observed in their expression levels.

### 3.5 Therapeutic effects of NK cells combined with Bev plus Iri in the s.c U87 xenograft mouse model

**Figure 3:**
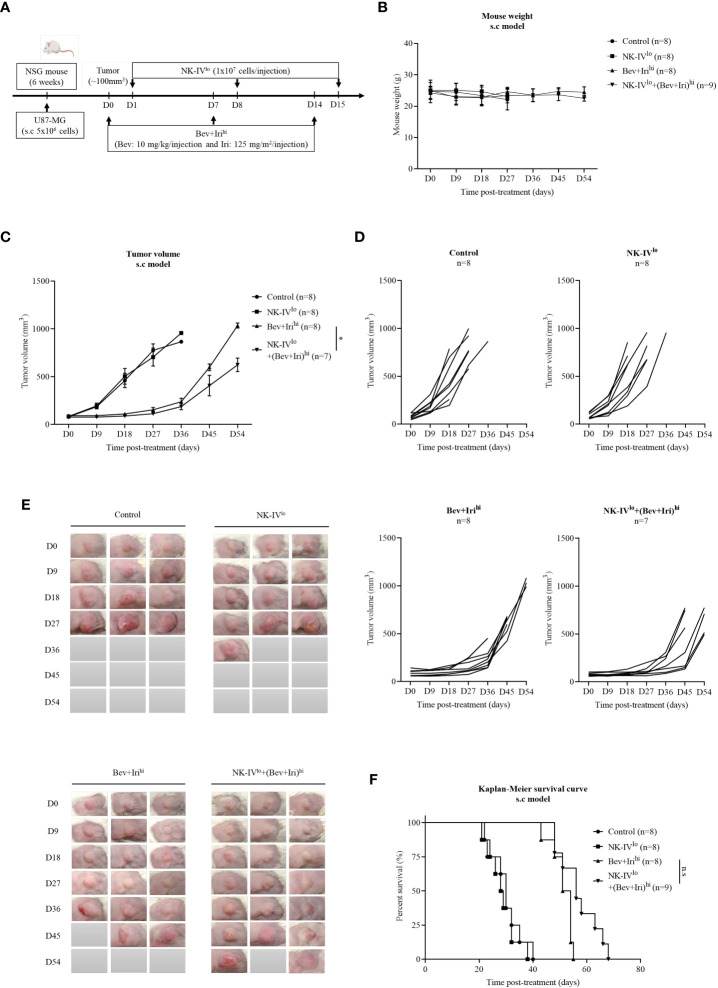
Experimental treatment schedules and therapeutic effects of NK-HD cells in combination with Bev plus Iri estimated in the s.c U87 mouse model. At a tumor size of 100 mm^3^, the mice were treated with Bev and Iri every 7 days for 14 days. NK-HD cells were injected after 1 day of Bev and Iri treatment **(A)**. The mouse weight before and after the treatment with NK-HD cells in combination with Bev and Iri was clarified every 9 days **(B)**. The tumor size before and after the treatment with NK-HD cells in combination with Bev and Iri was clarified every 9 days and the tumor volume was calculated as follows: V = 4/3π (length × width × height/8) **(C–E)**. The Kaplan–Meier survival of U87-bearing mice of control, NK-IV^lo^, Bev plus Iri^hi^, and NK-IV^lo^ in combination with Bev plus Iri^hi^ group was estimated. The statistical significance of survival was determined using a log-rank test with Bonferroni correction applied for comparisons **(F)**. NK-IV^lo^: intravenously injected NK-HD cells (1 × 10^7^ NK cells); Bev+Iri^hi^: intraperitoneally injected bevacizumab (10 mg/kg) plus irinotecan (125 mg/m^2^); U87: human GBM cell line. All data are shown as mean ± SD. p< 0.05 (*). n.s, no significant difference.

When the tumor reached around 100 mm^3^, U87-bearing mice were treated with NK-HD cells, Bev, and Iri according to the scheduled treatment shown in **Figure 3A**. Days were estimated from post-treatment. Further, mouse weight was estimated; as shown in **Figure 3B**, there was no difference in mouse weight between treatment groups such as NK-IV^low^, Bev plus Iri^high^, and NK-IV^low^ combined with Bev plus Iri^high^ group compared to the control group. The therapeutic effects of NK-HD cells combined with Bev plus Iri were confirmed from tumor volume and survival.

The tumor volume was also measured with a digital caliper; the final data are shown in **Figures 3C–E**; **S5**. As revealed by the aforementioned figures, there was no difference in tumor volume between NK-IV^low^ and control groups. However, the tumor volume of the mice treated with Bev plus Iri^high^ and NK-IV^low^ combined with Bev plus Iri^high^ showed a delay in tumor growth compared to the control and NK-IV^low^ groups. Bev plus Iri^high^ and NK-IV^low^ combined with Bev plus Iri^high^ reduced the tumor volume compared with that of the control group (p = 0.000 and p = 0.000, respectively) and NK-IV^low^ (p = 0.000 and p = 0.000, respectively). Moreover, NK-IV^low^ combined with Bev plus Iri^high^ also showed suppressed tumor growth compared with the Bev plus Iri^high^ group (p = 0.037).

Similarly, the survival of U87-bearing mice did not differ between NK-IV^low^ (28.8 ± 1.7 days) and the control group (30 ± 2.1 days). However, the survival of the mice treated with Bev plus Iri^high^ and NK-IV^low^ combined with Bev plus Iri^high^ significantly differed from that of the control and NK-IV^low^ groups. Bev plus Iri^high^ (51.3 ± 1.4 days) and NK-IV^low^ combined with Bev plus Iri^high^ (57.1 ± 2.5 days) prolonged the survival of the treated mice compared with that of the control group (p = 0.000 and p = 0.000, respectively) and the mice treated with NK-IV^low^ (p = 0.000 and p = 0.000, respectively). Although there was a difference in mouse survival between Bev plus Iri^high^ and NK-IV^low^ combined with Bev plus Iri^high^, as revealed by the log-rank test (p = 0.018), there was no difference with the Bonferroni correction applied (**Figure 3F**).

### 3.6 Therapeutic effects of NK cells combined with Bev plus Iri in the i.c U87 xenograft mouse model

**Figure 4:**
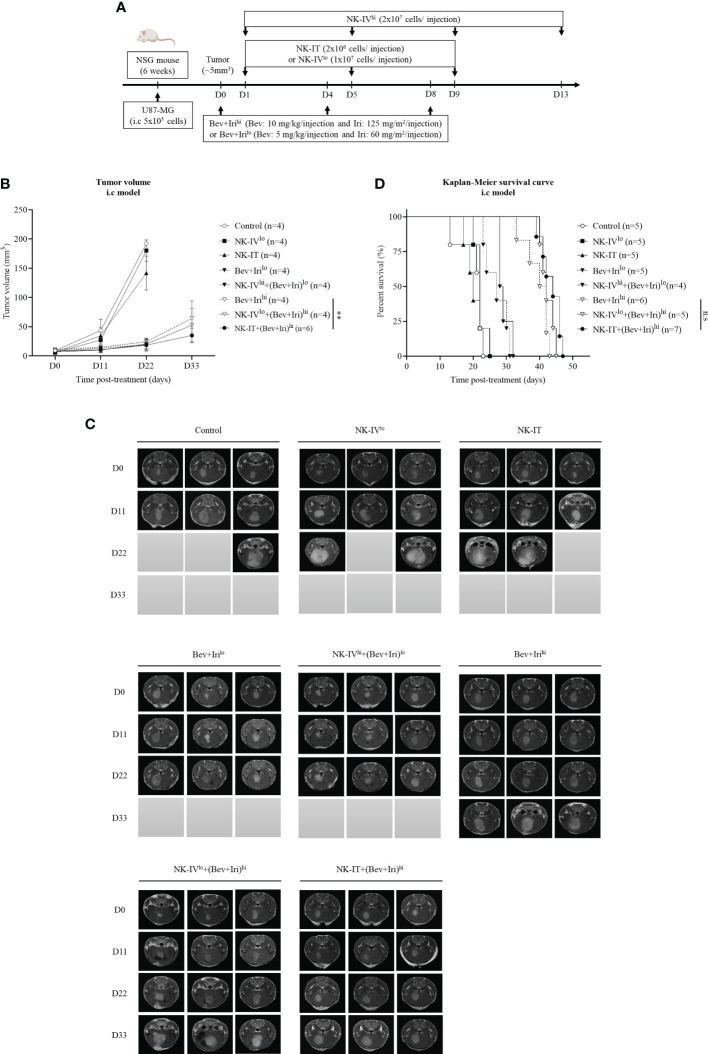
Experimental treatment schedule and therapeutic effects of NK-HD cells in combination with Bev plus Iri in the i.c U87 mouse model. At a tumor size of 5 mm^3^, the mice were treated with Bev and Iri every 4 days for 8 days. NK-HD cells were injected after 1 day of Bev and Iri treatment **(A)**. The tumor size before and after the treatment with NK-HD cells in combination with Bev plus Iri was clarified every 6 days using MRI. The final tumor volume was analyzed using the RadiAnt DICOM Viewer 2021.2.2 software and calculated as the summation of all tumor areas in each slide and multiplication by the slide thickness **(B, C)**. The Kaplan–Meier survival of U87-bearing mice after treatment with NK-HD cells in combination with Bev plus Iri was estimated. The statistical significance of survival was determined using a log-rank test with Bonferroni correction applied for comparisons **(D)**. NK-IV^lo^: intravenously injected NK-HD cells (1 × 10^7^ NK cells); NK-IV^hi^: intravenously injected NK-HD cells (2 × 10^7^ NK cells); Iri^hi^: intraperitoneally injected Iri (125 mg/m^2^); Bev+Iri^lo^: intraperitoneally injected Bev (5 mg/kg) plus Iri (60 mg/m^2^); Bev+Iri^hi^: intraperitoneally injected Bev (10 mg/kg) plus Iri (125 mg/m^2^); NK-IT: intratumorally injected NK-HD cells (2 × 10^6^ NK cells); U87: human GBM cell line. All data are shown as mean ± SD. p< 0.001 (**). n.s, no significant difference.

When the tumor reached around 5 mm^3^, U87-bearing mice were treated with NK-HD cells, Bev, and Iri according to the scheduled treatment in **Figure 4A**. The therapeutic effects of NK-HD cells combined with Bev plus Iri were confirmed in terms of tumor volume and survival. Days were estimated from post-treatment.

Tumor volume was clarified *via* MRI and mouse weight was also estimated (**Figures 4B, C**; **S6**, **S7**). The tumor volume of U87-bearing mice did not differ between NK-IV^low^ and NK-IT compared with that of the control group. However, the tumor volumes of the mice treated with Bev plus Iri^low^, NK-IV^high^ combined with Bev plus Iri^low^, Bev plus Iri^high^, NK-IV^low^ combined with Bev plus Iri^high^, and NK-IT combined with Bev plus Iri^high^ significantly differed from those of the control (p = 0.000, p = 0.000, p = 0.001, p = 0.000, and p = 0.000, respectively), NK-IV^low^ (p = 0.000, p = 0.000, p = 0.000, p = 0.000, and p = 0.000, respectively), and NK-IT (p = 0.000, p = 0.000, p = 0.001, p = 0.000, and p = 0.000, respectively) groups. Only NK-IT combined with Bev plus Iri^high^ delayed tumor growth compared with the Bev plus Iri^high^ group (p = 0.006). Moreover, Bev plus Iri^high^ also showed delayed tumor volume compared with Iri^high^ (p = 0.043). These results show the role of the combination of bevacizumab and irinotecan in the i.c U87 mouse model. The combination of NK-IV^high^ with Bev plus Iri^high^ could not suppress the tumor size compared to Bev plus Iri^high^.

A similar trend can be observed in **Figure 4D**, wherein the survival of U87-bearing mice did not differ between NK-IT and NK-IV^low^ compared with that of the control group (20.2 ± 1.8 days). However, Bev plus Iri^low^ (27.0 ± 1.6 days) and NK-IV^high^ combined with Bev plus Iri^low^ (29.3 ± 0.9 days) prolonged the survival of the treated mice compared with those of the control (p = 0.03 and p = 0.002, respectively), NK-IV^low^ (n.s and p = 0.038, respectively), and NK-IT (p = 0.03 and p = 0.002, respectively). Moreover, the survival of U87-bearing mice in Bev plus Iri^high^ (39.5 ± 1.6 days), NK-IV^low^ combined with Bev plus Iri^high^ (42.4 ± 0.9 days), and NK-IT combined with Bev plus Iri^high^ (43.6 ± 1.1 days) was also prolonged compared with that of the mice treated with Bev plus Iri^low^ (p = 0.000, p = 0.000, and p = 0.000, respectively) and NK-IV^high^ combined with Bev plus Iri^low^ (p = 0.000, p = 0.000, and p = 0.000, respectively). However, no difference in survival was observed between Bev plus Iri^high^, NK-IV^low^ combined with Bev plus Iri^high^, and NK-IT combined with Bev plus Iri^high^. The combination of NK-IV^high^ with Bev plus Iri^high^ affected mouse survival during treatment. However, the remaining mice recovered after 7 days post-treatment, and alive mice in NK-IV^high^ with Bev plus Iri^high^ group died owing to increased tumor volume.

### 3.7 Tumor-infiltrating NK cells and NK ligand expression in tumors

**Figure 5:**
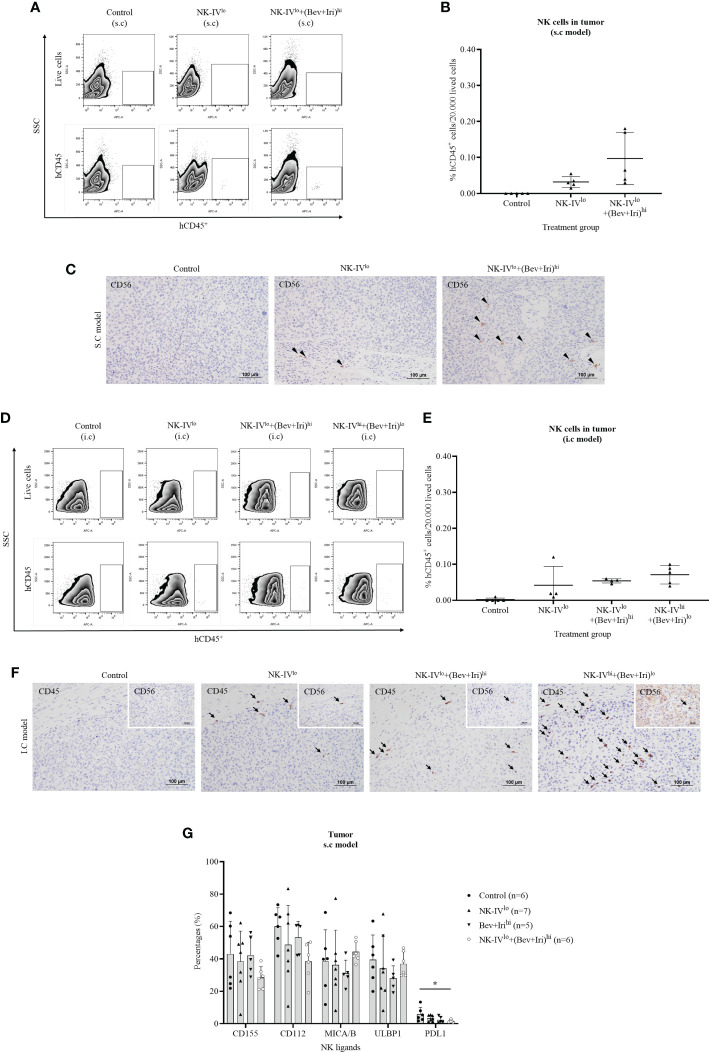
Presence of NK-HD cells in the tumor of s.c and i.c U87-bearing mice with or without treatment of NK-HD cells alone or NK-HD cells in combination with Bev plus Iri was confirmed through flow cytometry by hCD45 and immunohistochemistry (IHC) with hCD45 and hCD56 **(A–F)**. The expression levels of NK ligands such as CD155, CD112, MICA/B, ULBP1, and PDL1 on single tumor cells with or without treatment of NK-HD cells alone or NK cells in combination with Bev plus Iri were also clarified using flow cytometry **(G)**. Data are presented by percentages of positive cells. FMO: live cells without staining NK ligand markers; NK-IV^lo^: intravenously injected NK-HD cells (1 × 10^7^ NK cells); NK-IV^hi^: intravenously injected NK-HD cells (2 × 10^7^ NK cells); Bev+Iri^lo^: intraperitoneally injected Bev (5 mg/kg) plus Iri (60 mg/m^2^); Bev+Iri^hi^: intraperitoneally injected Bev (10 mg/kg) plus Iri (125 mg/m^2^); U87: human GBM cell line. All data are shown as mean ± SD. p< 0.05 (*).

The tumor-infiltrating NK-HD cells of U87-bearing mice were confirmed *via* flow cytometry with hCD45 and IHC with hCD56 and hCD45 markers; the results are presented in **Figures 5A–F**. The percentages of hCD45^+^ cells in tumors were clarified on the s.c and i.c U87 mouse models. In general, a few hCD45^+^ cells were detected in the tumor in the NK treatment group (NK-IV^low^) and NK cells in combination with Bev plus Iri group (NK-IV^low^ combined Bev plus Iri^high^ or NK-IV^high^ combined Bev plus Iri^low^) in both s.c and i.c U87 mouse models, which was confirmed with the presence of hCD45 and hCD56 in the tumor with IHC assay. Although NK-IV^low^ combined Bev plus Iri^high^ showed a slight enhancement compared to NK-IV^low^, no difference was observed in the s.c and i.c U87 mouse models. Moreover, the presence of NK cells in NK-IV^high^ combined with Bev plus Iri^low^ was confirmed by flow cytometry and IHC in the i.c U87 mouse model.

The expression of NK ligands on single tumor cells in the s.c U87 mouse model is illustrated in **Figures 5G**; **S8**. The expression of NK ligands (CD155, CD112, MICA/B, and ULBP1) on single tumor cells in the s.c model did not vary among the control, NK-IV^low^, Bev plus Iri^high^, and NK-IV^low^ combined with Bev plus Iri^high^ groups. However, NK-IV^low^ combined with Bev plus Iri^high^ reduced the PDL1 expression compared with that of the control group in the s.c U87 mouse model (p=0.041).

## 4 Discussion

Scientific reports and clinical studies have demonstrated the promising effects of NK cell therapy on cancer, particularly in GBM (34–37) (**Table 2**). However, NK cell function is frequently impaired in solid tumors. One of the reasons for this occurrence is that the solid tumor-associated microenvironment often becomes hypoxic and induces TGF-β, which promotes the downregulation of activated NK cell receptors in patients with cancer; consequently, their killing activity that targets tumors is impaired (38). Among various approaches, adoptive NK therapy, which involves *ex vivo* expanded and activated NK cells has emerged as a promising solution to overcome immunosuppression commonly observed in solid tumors by increasing the number and antitumor activity of NK cells (39). Therefore, we used NK cells that were expanded and activated according to the previously established protocol by using a feeder cell, K562 expressing the OX40 ligand and membrane-bound IL-18 and IL-21, which showed potential effects on multiple myeloma in the previous study (23); we wanted to confirm the therapeutic effects of these established NK cells in the GBM setting. Interestingly, NK-GBM showed no difference compared to NK-HDs in terms of purity, expansion fold, activated markers, and slightly lower cytotoxic effects against GBM cells with this established protocol.

**Table 2:** List of completed and ongoing clinical trials of NK cell therapy on GBM was obtained from https://www.clinicaltrials.gov (keywords: glioblastoma and NK cells) until November 2022.

No.	Study name	Identifier	Condition or disase	Treatment	Study phase	Description	Status	Starting date
I	Intracrani al Injection of NK-92/5.28.z Cells in Patients With Recurrent HER2- posit ive Glioblastoma (CAR2BRATN)	NCT03383978	• Glioblastoma	• NK-92/5.28.z	Phase I	Multicenter, Open Label, Phase I Study of I ntracranial I njection of NK-92/5.28.z Cells in Patients With Recurrent HER2- positive Glioblastoma	Recruiting	December 2017
2	NK Cell Therapy for Recurrent Glioblastoma Multifom1 Patients	NCT05108012	• Glioblastoma Multiforme • Recurrent Glioblastoma	• NK cell therapy	Phase I	The Safety Evaluation of Ex Vivo Activated Haploidentical Natural Killer Cells (NK) in Recurrent Glioblastoma Mu ltifonn Patients (Clinical Trial Phase I )	Recruiting	August 2021
3	Intra-tumoral Inject ion of Natural Killer Cells in High- Grade Gliomas (NK HGG)	NCT04254419	• High Grade Glioma	• NKcells	Phase I	Phase I Study of Intra- Tumoral Injections of Autologous Ex Vivo Expanded Natural Killer Cells in Children With Recurrent High- Grade Glioma	Not yet recruiting	July 2022
4	Engineered NK Cells Containing Deleted TGF-BetaR2 and NR3C1 for the Treatment of Recurrent Glioblastoma	NCT04991870	• Recurrent Gliosarcom a • Recurrent Supratentorial Glioblastom a • Supratentorial Gliosarcoma	• Cord Blood-derived Expanded Allogeneic Natural Killer Cells	Phase I	This phase I trial is to find out the best dose.possible benefits and/or side effects of engineered natural killer (NK) cells containing deleted TGF-betaR2 and NR3C1 (cord blood [CBJ- NK-TGF-betaR2-/NR3C1 -) in treating patients with glioblastoma that has come back (recurrent). CB- K- TGF-betaR2- /NR3C1 - cells are genetically changed immune cells that may help to control the disease.	Not yet recruiting	August 2022

All data were collected from studies with only NK cell treatment. Data from withdrawn, terminated, and suspended studies were excluded from the final summary.

The combination of different treatment methods to target tumors with different mechanisms aids in overcoming the high degree of heterogeneity in solid tumors. Specifically, combination therapy of different targeted drugs with adoptively transferred NK cells helps to improve the therapeutic effects against solid tumors (40). Among different drugs, Bev and Iri have been studied separately or in combination in clinical trials for treating GBM (11, 41) (**Table 3**). Although Bev did not affect the proliferation of GBM cells *in vitro* in a previous study (42), the combination of anti-angiogenic drugs (Bev) with NK cell therapies was found to boost NK cell infiltration into the solid tumor, thereby improving efficacy (43). A trial combining bevacizumab and allogeneic NK therapy is investigating this combination approach (NCT02857920). Moreover, Bev in combination with Iri enhances the function of irinotecan through the normalization of tumor vasculature; therefore, the delivery of irinotecan into tumors is increased. A combination of Bev and Iri showed an active regimen with acceptable toxicity in recurrent malignant gliomas (44). In addition, the combination of Bev and Iri delayed tumor growth compared to Iri alone in the i.c U87 mouse model in a previous study (45). In our study, although Bev also did not affect the proliferation and NK ligand expression of GBM cells *in vitro*, Bev combined with Iri delayed tumor growth compared to Iri alone. A combination of Bev and Iri also reduced tumor volume and extended survival in these s.c and i.c U87 mouse models. We also clarified the role of Iri in increasing the cytotoxicity of NK cells against GBM cells through enhancement of the MICA/B expression; however, we were unable to find the relationship between this toxicity related to released IFN-γ and NK marker expression after co-culturing with Bev plus Iri-treated GBM cells. Although this is the first study to highlight the potential for using NK cells combined with Bev and Iri, a more detailed role of NK cells, Bev, and Iri in this combination should be investigated.

**Table 3:** A list of completed and ongoing clinical trials of combination treatment of Bev and Iri on GBM was obtained from www.clinicaltrials.gov (keywords: glioblastoma, bevacizumab, and irinotecan) until November 2022.

No.	Study name	Identifier	Condition or disease	Treatment	Study Phase	Description	Status	Starting date
1	A Study to Evaluate Bevacizumab Alone or in CombinationWith Irinotecan for Treatmentof Glioblastoma Multiforme (BRAIN) (BRAIN)	NCT00345163	Glioblastoma	- Bevacizumab- Irinotecan	Phase II	A Phase II, Multicenter, Randomized, Non- Comparative Clinical Trial to Evaluate the Efficacy and Safety of Bevacizumab Alone or in Combination With Irinotecan for Treatment of Glioblastoma Multiforme in First or Second Relapse	Completed	July 2006
2	A Study to Evaluate the Efficacy of Bevacizumab Plus Irinotecan in Recurrent Gliomas	NCT00921167	- Glioblastoma- Astrocytoma	- Bevacizumab- Irinotecan	Phase II	A Phase II Study to Evaluate the Efficacy of BevacizumabPlus Irinotecan in Recurrent Anaplastic Astrocytoma or Recurrent Glioblastoma Multiforme	Completed	June2009

All data were collected from studies with only bevacizumab combined with irinotecan treatment. Data from withdraw, terminated, and suspended studies were excluded from the final summary.

U87 cells, which are implanted into immunodeficient mice such as nude, NOD/SCID, and NOD/SCID gamma (NSG), are widely employed and have proved useful in the assessment of GBM angiogenesis and anti-angiogenic therapeutic approaches (46–48). In this study, the U87 mouse model was also used to confirm the therapeutic effects of NK cells in combination with Bev and Iri. However, the lack of NK cells plus Bev resulted in weak evidence to confirm the synergistic effects of Bev in enhancing NK cell infiltration. Although U87 tumors are known to lack tumor infiltration (48), a low level of macrophages and lymphocytes are still able to infiltrate U87 human GBM xenografts in nude mice, as reported in a previous study (49). In agreement with this previous finding, in the present study, the NK cells detected in tumors were very low in numbers in both s.c and i.c models by IV injection of NK cells. However, we did not clarify that low levels of NK cells infiltrated the U87 tumor after treatment due to the low migration and infiltration of NK cell target tumor or NK cells in the tumor and their death after performing a function when they were detected. More experiments should be conducted to highlight this issue using another xenograft mouse model, which displays a highly infiltrative and invasive growth pattern like U251 tumors or the GBM murine model.

Our study highlights the potential use of adoptive transfer NK cells in combination with Bev and Iri by delaying tumor growth in both s.c and i.c U87 mouse models. We showed that Iri alone has short-term effects on U87 tumors owing to the regrowth of tumors post-treatment. However, Bev combined with Iri delayed tumor growth compared to Iri alone. Moreover, NK cells combined with Bev and Iri showed slow tumor growth compared to Bev plus Iri. Thus, NK cells have targeted resistance with Bev and Iri cells. However, this combination failed to improve mouse survival. Further, NK cells have been reported to contribute to antitumor immunity not only by directly eliminating malignant cells but also by regulating tumor-specific adaptive immune response through crosstalk with dendritic cells (50). However, the complete function of NK cell therapy alone or in combination with Bev and Iri was not examined in a complete human system.

## 5 Conclusion

Although the NK cells that were expanded by using K562-OX40L-mb-IL-18/IL-21 feeder cells showed cytotoxic effects against GBM cells *in vitro* and no therapeutic effects on single treatment with s.c and i.c U87 GBM models, the combination of NK cells with Bev and Iri enhanced cytotoxic effects against GBM cells *in vitro* and delayed tumor progression during early-stage treatment. However, the NK cell combination with Bev plus Iri was unable to improve survival in both s.c and i.c U87 GBM models with this setting. Therefore, other settings should be considered to highlight the synergistic effects among NK cells, Bev, and Iri.

## Data availability statement

The original contributions presented in the study are included in the article/**Supplementary Material**. Further inquiries can be directed to the corresponding authors.

## Ethics statement

The studies involving human participants were reviewed and approved by Institutional Review Board of Chonnam National University Hwasun Hospital. The patients/participants provided their written informed consent to participate in this study. The animal study was reviewed and approved by Chonnam National University Animal Research Committee.

## Author contributions

T-A-TT, Y-HK, K-HL, and T-YJ designed and performed the experiments. T-A-TT, Y-HK, T-H-OD, K-HL, and T-YJ analyzed and presented the data. T-A-TT and T-YJ wrote the article. JT and T-HC contributed to the preliminary experiments. SJ, I-YK, K-SM, Y-JK, T-KL, J-JL, CL, HY, K-HL, and T-YJ contributed intellectually to the research. All authors contributed to the article and approved the submitted version.

## References

[1] Ostrom QT Cioffi G Waite K Kruchko C Barnholtz-Sloan JS. CBTRUS statistical report: Primary brain and other central nervous system tumors diagnosed in the united states in 2014-2018. Neuro Oncol (2021) 23(12 Suppl 2):iii1–iii105. doi: 10.1093/neuonc/noab200 34608945 PMC8491279

[2] Miller KD Ostrom QT Kruchko C Patil N Tihan T Cioffi G. Brain and other central nervous system tumor statistics, 2021. CA Cancer J Clin (2021) 71(5):381–406. doi: 10.3322/caac.21693 34427324

[3] Stupp R Hegi ME Mason WP van den Bent MJ Taphoorn MJ Janzer RC. Effects of radiotherapy with concomitant and adjuvant temozolomide versus radiotherapy alone on survival in glioblastoma in a randomised phase III study: 5-year analysis of the EORTC-NCIC trial. Lancet Oncol (2009) 10(5):459–66. doi: 10.1016/S1470-2045(09)70025-7 19269895

[4] Ostrom QT Cote DJ Ascha M Kruchko C Barnholtz-Sloan JS. Adult glioma incidence and survival by race or ethnicity in the united states from 2000 to 2014. JAMA Oncol (2018) 4(9):1254–62. doi: 10.1001/jamaoncol.2018.1789 PMC6143018 29931168

[5] Krcek R Matschke V Theis V Adamietz IA Buhler H Theiss C. Vascular endothelial growth factor, irradiation, and axitinib have diverse effects on motility and proliferation of glioblastoma multiforme cells. Front Oncol (2017) 7:182. doi: 10.3389/fonc.2017.00182 28879167 PMC5572260

[6] Ferrara N. VEGF and intraocular neovascularization: From discovery to therapy. Transl Vis Sci Technol (2016) 5(2):10. doi: 10.1167/tvst.5.2.10 PMC4790412 26981332

[7] Mukwaya A Jensen L Lagali N. Relapse of pathological angiogenesis: functional role of the basement membrane and potential treatment strategies. Exp Mol Med (2021) 53(2):189–201. doi: 10.1038/s12276-021-00566-2 33589713 PMC8080572

[8] Al-Ostoot FH Salah S Khamees HA Khanum SA. Tumor angiogenesis: Current challenges and therapeutic opportunities. Cancer Treat Res Commun (2021) 28:100422. doi: 10.1016/j.ctarc.2021.100422 34147821

[9] Haibe Y Kreidieh M El Hajj H Khalifeh I Mukherji D Temraz S. Resistance mechanisms to anti-angiogenic therapies in cancer. Front Oncol (2020) 10:221. doi: 10.3389/fonc.2020.00221 32175278 PMC7056882

[10] Cohen MH Shen YL Keegan P Pazdur R. FDA Drug approval summary: Bevacizumab (Avastin (R)) as treatment of recurrent glioblastoma multiforme. Oncologist (2009) 14(11):1131–8. doi: 10.1634/theoncologist.2009-0121 19897538

[11] Vredenburgh JJ Desjardins A Reardon DA Friedman HS. Experience with irinotecan for the treatment of malignant glioma. Neuro Oncol (2009) 11(1):80–91. doi: 10.1215/15228517-2008-075 18784279 PMC2718962

[12] Cloughesy TF Filka E Kuhn J Nelson G Kabbinavar F Friedman H. Two studies evaluating irinotecan treatment for recurrent malignant glioma using an every-3-week regimen. Cancer-Am Cancer Soc (2003) 97(9):2381–6. doi: 10.1002/cncr.11306 12712460

[13] Cloughesy TF Filka E Nelson G Kabbinavar F Friedman H Miller LL. Irinotecan treatment for recurrent malignant glioma using an every-3-week regimen. Am J Clin Oncol-Canc (2002) 25(2):204–8. doi: 10.1097/00000421-200204000-00022 11943904

[14] Lu G Rao M Zhu P Liang B El-Nazer RT Fonkem E. Triple-drug therapy with bevacizumab, irinotecan, and temozolomide plus tumor treating fields for recurrent glioblastoma: A retrospective study. Front Neurol (2019) 10:42. doi: 10.3389/fneur.2019.00042 30766509 PMC6366009

[15] Moller S Grunnet K Hansen S Schultz H Holmberg M Sorensen M. A phase II trial with bevacizumab and irinotecan for patients with primary brain tumors and progression after standard therapy. Acta Oncol (2012) 51(6):797–804. doi: 10.3109/0284186X.2012.681063 22548369

[16] Ali SA McHayleh WM Ahmad A Sehgal R Braffet M Rahman M. Bevacizumab and irinotecan therapy in glioblastoma multiforme: a series of 13 cases. J Neurosurg (2008) 109(2):268–72. doi: 10.3171/JNS/2008/109/8/0268 18671639

[17] Gururangan S Fangusaro J Poussaint TY McLendon RE Onar-Thomas A Wu S. Efficacy of bevacizumab plus irinotecan in children with recurrent low-grade gliomas–a pediatric brain tumor consortium study. Neuro Oncol (2014) 16(2):310–7. doi: 10.1093/neuonc/not154 PMC3895377 24311632

[18] Vredenburgh JJ Desjardins A Herndon JE Marcello J Reardon DA Quinn JA. Bevacizumab plus irinotecan in recurrent glioblastoma multiforme. J Clin Oncol (2007) 25(30):4722–9. doi: 10.1200/JCO.2007.12.2440 17947719

[19] Goel S Duda DG Xu L Munn LL Boucher Y Fukumura D. Normalization of the vasculature for treatment of cancer and other diseases. Physiol Rev (2011) 91:1071. doi: 10.1152/physrev.00038.2010 21742796 PMC3258432

[20] Zhang CC Burger MC Jennewein L Genssler S Schonfeld K Zeiner P. ErbB2/HER2-specific NK cells for targeted therapy of glioblastoma. Jnci-J Natl Cancer I (2016) 108(5):dj375. doi: 10.1093/jnci/djv375 26640245

[21] Melaiu O Lucarini V Cifaldi L Fruci D. Influence of the tumor microenvironment on NK cell function in solid tumors. Front Immunol (2019) 10:3038. doi: 10.3389/fimmu.2019.03038 32038612 PMC6985149

[22] Khatua S Cooper LJN Sandberg DI Ketonen L Johnson JM Rytting ME. Phase I study of intraventricular infusions of autologous ex vivo expanded NK cells in children with recurrent medulloblastoma and ependymoma. Neuro-Oncology (2020) 22(8):1214–25. doi: 10.1093/neuonc/noaa047 PMC7594549 32152626

[23] Thangaraj JL Ahn SY Jung SH Vo MC Chu TH Thi Phan MT. Expanded natural killer cells augment the antimyeloma effect of daratumumab, bortezomib, and dexamethasone in a mouse model. Cell Mol Immunol (2021) 18(7):1652–61. doi: 10.1038/s41423-021-00686-9 PMC8245645 33980993

[24] Thangaraj JL Jung SH Vo MC Chu TH Phan MT Lee KH. Expanded natural killer cells potentiate the antimyeloma activity of daratumumab, lenalidomide, and dexamethasone in a myeloma xenograft model. Cancer Immunol Immunother (2022). doi: 10.1007/s00262-022-03322-1 PMC10110729 36385211

[25] Thangaraj JL Phan MT Kweon S Kim J Lee JM Hwang I. Expansion of cytotoxic natural killer cells in multiple myeloma patients using K562 cells expressing OX40 ligand and membrane-bound IL-18 and IL-21. Cancer Immunol Immunother (2022) 71(3):613–25. doi: 10.1007/s00262-021-02982-9 PMC10991462 34282497

[26] Qazi MA Vora P Venugopal C Sidhu SS Moffat J Swanton C. Intratumoral heterogeneity: pathways to treatment resistance and relapse in human glioblastoma. Ann Oncol (2017) 28(7):1448–56. doi: 10.1093/annonc/mdx169 28407030

[27] Garcia J Hurwitz HI Sandler AB Miles D Coleman RL Deurloo R. Bevacizumab (Avastin (R)) in cancer treatment: A review of 15 years of clinical experience and future outlook. Cancer Treat Rev (2020) 86:102017. doi: 10.1016/j.ctrv.2020.102017 32335505

[28] Paul S Lal G. The molecular mechanism of natural killer cells function and its importance in cancer immunotherapy. Front Immunol (2017) 8:1124. doi: 10.3389/fimmu.2017.01124 28955340 PMC5601256

[29] Vredenburgh JJ Desjardins A Herndon JE 2nd Dowell JM Reardon DA Quinn JA. Phase II trial of bevacizumab and irinotecan in recurrent malignant glioma. Clin Cancer Res (2007) 13(4):1253–9. doi: 10.1158/1078-0432.CCR-06-2309 17317837

[30] Kreisl TN Kim L Moore K Duic P Royce C Stroud I. Phase II trial of single-agent bevacizumab followed by bevacizumab plus irinotecan at tumor progression in recurrent glioblastoma. J Clin Oncol (2009) 27(5):740–5. doi: 10.1200/JCO.2008.16.3055 PMC2645088 19114704

[31] Kenmotsu H Niho S Tsuboi M Wakabayashi M Ishii G Nakagawa K. Randomized phase III study of irinotecan plus cisplatin versus etoposide plus cisplatin for completely resected high-grade neuroendocrine carcinoma of the lung: JCOG1205/1206. J Clin Oncol (2020) 38(36):4292–301. doi: 10.1200/JCO.20.01806 33136471

[32] Gleeson JP Keane F Keegan NM Mammadov E Harrold E Alhusaini A. Similar overall survival with reduced vs. standard dose bevacizumab monotherapy in progressive glioblastoma. Cancer Med-Us (2020) 9(2):469–75. doi: 10.1002/cam4.2616 PMC6970030 31756059

[33] Nair AB Jacob S. A simple practice guide for dose conversion between animals and human. J Basic Clin Pharm (2016) 7(2):27–31. doi: 10.4103/0976-0105.177703 27057123 PMC4804402

[34] Liu S Galat V Galat Y Lee YKA Wainwright D Wu J. NK cell-based cancer immunotherapy: from basic biology to clinical development. J Hematol Oncol (2021) 14(1):7. doi: 10.1186/s13045-020-01014-w 33407739 PMC7788999

[35] Pan C Zhai Y Li G Jiang T Zhang W. NK cell-based immunotherapy and therapeutic perspective in gliomas. Front Oncol (2021) 11:751183. doi: 10.3389/fonc.2021.751183 34765554 PMC8576093

[36] Sedgwick AJ Ghazanfari N Constantinescu P Mantamadiotis T Barrow AD. The role of NK cells and innate lymphoid cells in brain cancer. Front Immunol (2020) 11:1549. doi: 10.3389/fimmu.2020.01549 32903717 PMC7438769

[37] Burger MC Zhang C Harter PN Romanski A Strassheimer F Senft C. CAR-engineered NK cells for the treatment of glioblastoma: Turning innate effectors into precision tools for cancer immunotherapy. Front Immunol (2019) 10:2683. doi: 10.3389/fimmu.2019.02683 31798595 PMC6868035

[38] Chambers AM Lupo KB Matosevic S. Tumor microenvironment-induced immunometabolic reprogramming of natural killer cells. Front Immunol (2018) 9:2517. doi: 10.3389/fimmu.2018.02517 30467503 PMC6235907

[39] Shin MH Kim J Lim SA Kim J Kim SJ Lee KM. NK cell-based immunotherapies in cancer. Immune Netw (2020) 20(2):e14. doi: 10.4110/in.2020.20.e14 32395366 PMC7192832

[40] Li L Li W Wang C Yan X Wang Y Niu C. Adoptive transfer of natural killer cells in combination with chemotherapy improves outcomes of patients with locally advanced colon carcinoma. Cytotherapy (2018) 20(1):134–48. doi: 10.1016/j.jcyt.2017.09.009 29056549

[41] Agha CA Ibrahim S Hassan A Elias DA Fathallah-Shaykh HM. Bevacizumab is active as a single agent against recurrent malignant gliomas. Anticancer Res (2010) 30(2):609–11. 20332478

[42] von Baumgarten L Brucker D Tirniceru A Kienast Y Grau S Burgold S. Bevacizumab has differential and dose-dependent effects on glioma blood vessels and tumor cells. Clin Cancer Res (2011) 17(19):6192–205. doi: 10.1158/1078-0432.CCR-10-1868 21788357

[43] Xu C Liu D Chen Z Zhuo F Sun H Hu J. Umbilical cord blood-derived natural killer cells combined with bevacizumab for colorectal cancer treatment. Hum Gene Ther (2019) 30(4):459–70. doi: 10.1089/hum.2018.011 29914273

[44] Mesti T Moltara ME Boc M Rebersek M Ocvirk J. Bevacizumab and irinotecan in recurrent malignant glioma, a single institution experience. Radiol Oncol (2015) 49(1):80–5. doi: 10.2478/raon-2014-0021 PMC4362611 25810706

[45] Wang W Sivakumar W Torres S Jhaveri N Vaikari VP Gong A. Effects of convection-enhanced delivery of bevacizumab on survival of glioma-bearing animals. Neurosurg Focus (2015) 38(3):E8. doi: 10.3171/2015.1.FOCUS14743 25727230

[46] Kirsch M Strasser J Allende R Bello L Zhang J Black PM. Angiostatin suppresses malignant glioma growth *in vivo* . Cancer Res (1998) 58(20):4654–9. 9788618

[47] Schmidt NO Ziu M Carrabba G Giussani C Bello L Sun Y. Antiangiogenic therapy by local intracerebral microinfusion improves treatment efficiency and survival in an orthotopic human glioblastoma model. Clin Cancer Res (2004) 10(4):1255–62. doi: 10.1158/1078-0432.CCR-03-0052 14977823

[48] Haddad AF Young JS Amara D Berger MS Raleigh DR Aghi MK. Mouse models of glioblastoma for the evaluation of novel therapeutic strategies. Neurooncol Adv (2021) 3(1):vdab100. doi: 10.1093/noajnl/vdab100 34466804 PMC8403483

[49] Candolfi M Curtin JF Nichols WS Muhammad AG King GD Pluhar GE. Intracranial glioblastoma models in preclinical neuro-oncology: neuropathological characterization and tumor progression. J Neurooncol (2007) 85(2):133–48. doi: 10.1007/s11060-007-9400-9 PMC2384236 17874037

[50] Bodder J Zahan T van Slooten R Schreibelt G de Vries IJM Florez-Grau G. Harnessing the cDC1-NK cross-talk in the tumor microenvironment to battle cancer. Front Immunol (2021) 11:631713. doi: 10.3389/fimmu.2020.631713 33679726 PMC7933030

