# Supplementary figures and images for: Natural killer cell therapy potentially enhances the antitumor effects of bevacizumab plus irinotecan in a glioblastoma mouse model
Source: Front Immunol. 2023 Jan 10;13:1009484. doi: 10.3389/fimmu.2022.1009484 (PMC9871756; doi:10.3389/fimmu.2022.1009484)

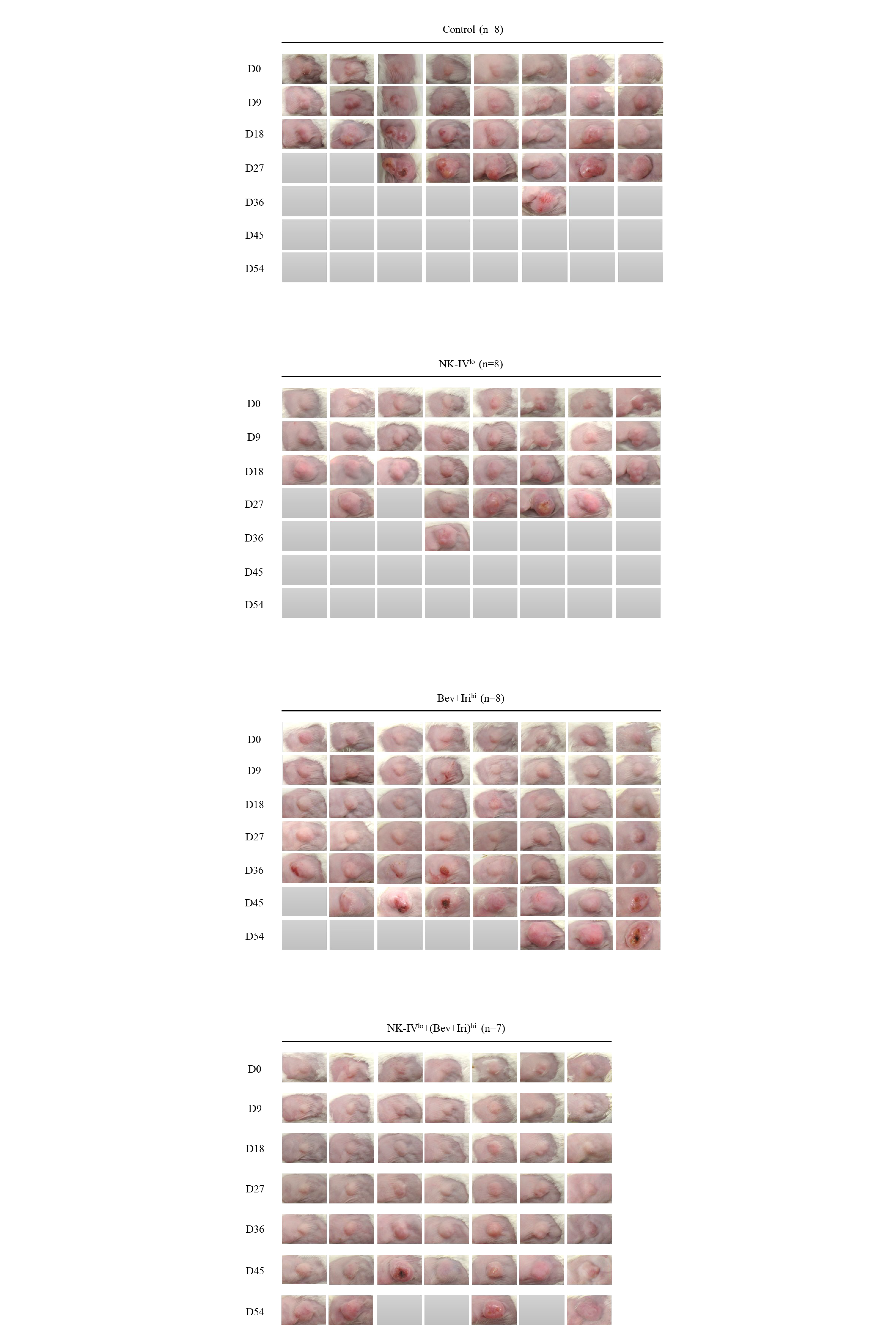

Supplement: Supplementary file 1 [file DataSheet_1.zip › Supplementary figure 5.TIFF]
